# The Relationship between Body Mass Index and the Severity of Coronary Artery Disease in Patients Referred for Coronary Angiography

**DOI:** 10.1155/2017/5481671

**Published:** 2017-04-23

**Authors:** Anne B. Gregory, Kendra K. Lester, Deborah M. Gregory, Laurie K. Twells, William K. Midodzi, Neil J. Pearce

**Affiliations:** ^1^Department of Clinical Epidemiology, Faculty of Medicine, Memorial University of Newfoundland, St. John's, NL, Canada A1B 3V6; ^2^Department of Medicine, Faculty of Medicine, Memorial University of Newfoundland, St. John's, NL, Canada A1B 3V6; ^3^School of Pharmacy, Memorial University of Newfoundland, St. John's, NL, Canada A1B 3V6; ^4^Eastern Health, St. John's, NL, Canada A1B 3V6

## Abstract

*Background and Aim*. Obesity is associated with an increased risk of cardiovascular disease and may be associated with more severe coronary artery disease (CAD); however, the relationship between body mass index [BMI (kg/m^2^)] and CAD severity is uncertain and debatable. The aim of this study was to examine the relationship between BMI and angiographic severity of CAD.* Methods*. Duke Jeopardy Score (DJS), a prognostic tool predictive of 1-year mortality in CAD, was assigned to angiographic data of patients ≥18 years of age (*N* = 8,079). Patients were grouped into 3 BMI categories: normal (18.5–24.9 kg/m^2^), overweight (25.0–29.9 kg/m^2^), and obese (≥30 kg/m^2^); and multivariable adjusted hazard ratios for 1-year all-cause and cardiac-specific mortality were calculated.* Results*. Cardiac risk factor prevalence (e.g., diabetes, hypertension, and hyperlipidemia) significantly increased with increasing BMI. Unadjusted all-cause and cardiac-specific 1-year mortality tended to rise with incremental increases in DJS, with the exception of DJS 6 (*p* < 0.001). After adjusting for potential confounders, no significant association of BMI and all-cause (HR 0.70, 95% CI .48–1.02) or cardiac-specific (HR 1.11, 95% CI .64–1.92) mortality was found.* Conclusions*. This study failed to detect an association of BMI with 1-year all-cause or cardiac-specific mortality after adjustment for potential confounding variables.

## 1. Introduction

Obesity is an independent risk factor for cardiovascular disease (CVD) [1–5] and is associated with advanced CVD requiring procedures such as percutaneous coronary intervention (PCI), reduction in life expectancy [6], and a higher mortality rate [3, 7, 8]. Weight loss has been associated with improvement in preexisting cardiovascular risk factors including hypertension (HTN), diabetes, and dyslipidemia and mortality [9–12]. Other studies have reported improved clinical outcomes in overweight and obese patients treated for CVDs compared to normal weight patients, suggesting a paradoxical survival benefit. This effect has been reported in patients with diabetes [13], end-stage renal disease [14], HTN [15], and other conditions traditionally associated with poorer outcomes [15–23]. Obesity was primarily measured using BMI in the studies. The mechanisms leading to this phenomenon, termed “obesity paradox,” are unclear.

The quantification of coronary artery disease (CAD) severity can be captured using coronary angiography (CA) [24]. Historically CAD has been categorized as single, double, and triple vessel and left main disease, with luminal stenosis of either ≥50% (left main) or ≥70% (other major epicardial vessels) used to define significance [25]. Scoring systems to determine the severity of CAD and prognosis were developed to address the perceived limitations associated with stratification of patients with risk level variation [26–28].

Few studies have examined the association of body mass index (BMI) and CAD in patients undergoing CA. In a study by Rubinshtein et al. [29] obese patients referred for CA were younger and had a lower prevalence of left main disease. Niraj et al. [30] also found that obese patients referred for CA were younger and had a lower burden for CAD; however, the authors did not find obesity to be a significant predictor for severity of CAD after adjustment for confounders suggesting that younger age may influence the obesity paradox. Others have reported an inverse relationship between BMI and severity of CAD in a cross-sectional, prospective study of 414 patients with suspected CAD [31].

Obesity is an accepted risk factor for CAD; therefore, it may be assumed that obese patients have poorer outcomes than nonobese patients [32]. However, published findings contradict this supposition about the relationship between BMI and mortality in patients undergoing CA for suspected CAD. The influence of BMI on extent of coronary atherosclerosis and cardiac events in a cohort of patients at risk of CAD was examined by Rossi et al. [33]. BMI was not significantly associated with extent of coronary atherosclerosis and mortality confirming the findings of others [29, 34, 35].

Newfoundland and Labrador (NL), a Canadian province, has the highest rate of obesity in the country and it is estimated that 71% of the province's population will be either overweight or obese by 2019 [36]. The aim of the current study was to examine the relationship between BMI and severity of CAD and determine what impact, if any, BMI had on 1-year mortality in the NL patient population referred for CA at a single tertiary care centre.

## 2. Methods

### 2.1. Setting, Study Design, and Data Collection

Secondary analysis of deidentified data for all patients ≥18 years of age that had CA between January 1, 2008, and December 31, 2012, in NL, Canada was conducted using a large population-based clinical database. A clinical software application (i.e., Alberta Provincial Project for Outcome Assessment in Coronary Heart Disease [APPROACH]) is used to prospectively collect detailed demographic, clinical, and procedural data on all patients referred for and undergoing CA and coronary revascularization procedures. Details of the database and methods of collection have been previously described [37].

Patients undergoing CA were identified from the APPROACH-NL database. There were 13936 diagnostic CAs performed from January 1, 2008, to December 31, 2012. Eligible subjects included all residents of NL ≥ 18 years with a BMI ≥ 18.5 kg/m^2^. The index CA and DJS were used; therefore, duplicate cases were excluded. The following patients were also excluded from the study: missing DJS data; missing BMI data or underweight; <18 years of age; missing indication code for CA or if the CA was performed for any reason other than the following: acute coronary syndrome, stable angina, unstable angina, atypical pain, serious arrhythmia, or presenting with cardiovascular symptoms not matching the above-mentioned common diagnostic categories. Since the focus of the study was on patients with suspected but not yet confirmed CAD, patients with established CAD (i.e., history of CABG, PCI, or myocardial infarction) were excluded from the study. After exclusion criteria were applied, a final study sample of 8,079 patients having a first CA for suspected, but not yet confirmed, CAD was identified.

Weight and height were measured and documented by a nurse at the time of CA. If patients were unstable, self-reported weight and height were collected and BMI calculated. Patients were grouped according to three BMI categories using the World Health Organization classification system: normal (18.5–24.9 kg/m^2^), overweight (25.0–29.9 kg/m^2^), and obese class > 30 kg/m^2^ [38] reflective of relative levels of risk to health [39]. Obese patients are much more likely to die from cardiac causes and lean patients are much more likely to die from noncardiac causes over a 10-year period following index myocardial infarction [40]. In the current study, the underweight BMI category (BMI < 18.5 kg/m^2^) was excluded because of the potential impact of comorbid conditions (e.g., advanced heart failure, cachexia) on outcome, conditions which are not captured in APPROACH-NL.

CA data were obtained from the Coronary Artery Reporting and Archiving Tool (CARAT), a graphic recording and communication application [41]. Detailed angiographic findings of all patients undergoing CA are automatically populated in APPROACH and a PDF file is created containing the anatomy of the coronary arteries according to the DJS [27] and becomes part of each patient's medical record. In the current study, severity and extent of obstructive CAD are based on the DJS. Dash et al. [27] developed the DJS, a prognostic tool predictive of 1-year mortality in patients with CAD, which was validated by Califf et al. [28] in 1985. The coronary tree is divided into 6 segments: the left anterior coronary artery (LAD), diagonal branches of the LAD, septal perforating branches, the circumflex coronary artery, obtuse marginal branches, and the posterior descending coronary artery. All segments with ≥75% stenosis, or ≥50% left main stenosis, are considered to be at risk. Each such segment is assigned 2 points. The maximum possible number of points is 12. A score from 0 to 12 is assigned to each CA based on the number of segments involved and automatically populated in APPROACH. The usefulness of the DJS as a simple score that is easy to use clinically as a prognostic tool has been confirmed in a large Canadian population cohort of >20,000 patients undergoing PCI or CABG [42]. Following PCI, there was no difference between DJSs 0 and 2; however, a stepwise increase in 1-year mortality with a DJS of >2 was found.

Mortality data stored in the NL Centre for Health Information (NLCHI) Mortality System was provided to the cardiac care program via a data linkage. The primary outcomes were 1-year all-cause and cardiac-specific mortality.

### 2.2. Ethical Considerations

All patients who had a CA during the time period under examination gave written, informed consent to the cardiac care program for data collection and follow-up observation after CA. The study was approved by the provincial Health Research Ethics Authority.

### 2.3. Data Analysis

Analyses are based on 8,079 patients with a BMI ≥ 18.5 kg/m^2^ undergoing CA for the first time. Continuous variables are reported as mean ± standard deviation and were compared using ANOVA. Categorical variables are reported as number (%) and were compared using chi-square tests. After the assumptions of survival analysis were met, time-to-event outcomes were analyzed using Kaplan-Meier survival techniques. The final enrollment date was December 31, 2012, and patients without events were censored on December 31, 2013, the final date for which mortality data was available. Survival curves were compared using the log rank test. All factors that could potentially influence survival were included (see characteristics in Table 1) in addition to BMI and DJS. Univariate and multivariate-adjusted Cox regression models were performed to identify predictors of 1-year mortality and compute crude and multivariate-adjusted hazards ratios and 95% confidence intervals as a measure of the relative risk of death at one year for increasing BMI categories. Normal weight was the referent group (18.5–24.9 kg/m^2^). Covariates included BMI, DJS, age, sex, HTN, diabetes, hyperlipidemia, smoking history, family history of premature CAD, left ventricular (LV) grade, peripheral vascular disease (PVD), chronic obstructive pulmonary disease (COPD), renal insufficiency, dialysis, chronic renal failure (CRF), congestive heart failure (CHF), and malignancy. A two-sided *p* value < 0.05 was considered statistically significant. In the model all independent variables were dichotomous with the exception of age and BMI. BMI was included both as a continuous variable [43] and as an ordinal variable. Obesity was defined as a BMI ≥ 30 kg/m^2^. All statistical analyses were performed using IBM SPSS Statistics for Windows, Version 22.0, Armonk, NY: IBM Corp.

## 3. Results

Baseline characteristics are presented in Table 1. Among 8,079 patients approximately 84% were overweight or obese: 1,297 (16.1%) had a normal BMI, 3,072 (38%) had a BMI indicating overweight, and 3,710 (45.9%) were classified as obese. The average weight in kilograms for the entire sample was 85.2 ± 17.8 and the average BMI was 30.3 ± 5.7. There were significant differences among BMI categories in terms of age, sex, HTN, diabetes, hyperlipidemia, and family history of premature CAD, COPD, PVD, and LV grade. Significantly higher proportions of males compared to females comprised all BMI categories. As expected, the prevalence of HTN, hyperlipidemia, and diabetes significantly increased with increasing BMI. Patients with obesity were significantly younger and had a higher rate of family history of CAD and COPD. Normal weight patients had a higher rate of PVD, renal insufficiency, dialysis, LV Grades III and IV, and lower rate of admission for acute coronary syndrome (ACS). BMI groups did not differ significantly with regard to smoking history, CRF, CVD, malignancy, or CHF. There were statistically significant differences in medications at time of referral for coronary angiography. Higher proportions of obese patients were taking ACE inhibitors, ARB antagonists, and CCB, and a lower proportion of obese patients were taking ticlopidine/clopidogrel compared to normal or overweight patients.

DJSs calculated during CA by BMI category are presented in Table 1. A score of 0, indicative of a normal angiogram or noncritical (<70%) stenosis in any of the coronary arteries, was assigned to 526 (40.6%) normal weight patients, 1,197 (39.0%) overweight patients, and 1,687 (45.5%) obese patients. Differences were observed among BMI categories and all DJS levels (*p* < 0.001), with the exception of DJS ≥ 10. Patients in the obese group tended to have lower scores indicating less CAD severity.

**Figure 1:**
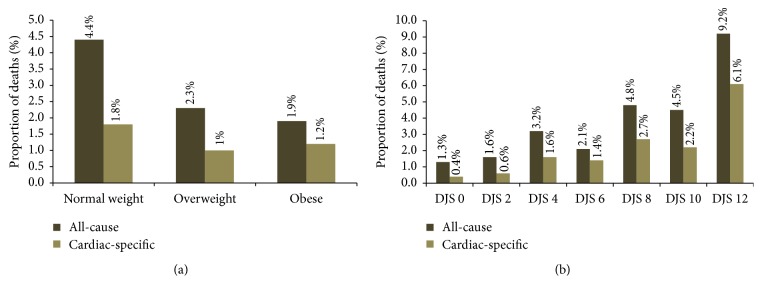
(a) Unadjusted 1-year all-cause and cardiac-specific mortality according to BMI. (b) Unadjusted 1-year all-cause and cardiac-specific mortality according to Duke Jeopardy Score.

Within the first year of undergoing CA there were 199 deaths (2.5%) among 8,079 patients, of which 99 (1.2%) were cardiac-specific. A higher proportion of deaths due to any cause occurred in patients with normal BMI compared to overweight or obese patients (*p* < 0.001); however, there were no statistically significant differences observed for unadjusted cardiac-specific mortality among BMI categories (Figure 1(a)). Unadjusted mortality tended to rise with incremental increases in DJS scores (*p* < 0.001), with the exception of DJS 6 (Figure 1(b)).

**Figure 2:**
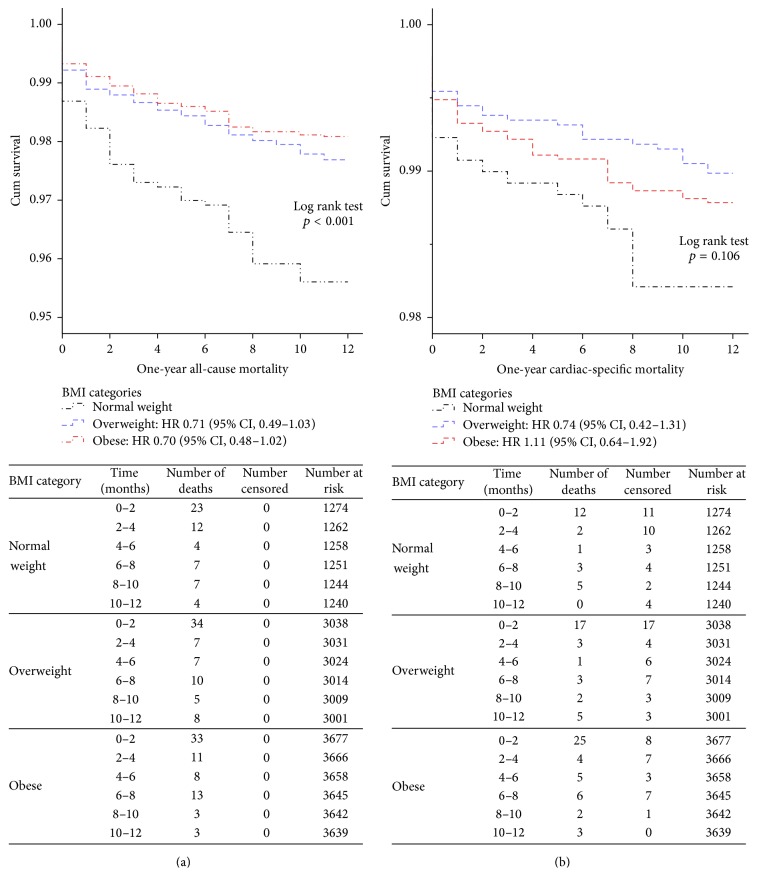
(a) Unadjusted Kaplan Meier and 1-year all-cause mortality in patients undergoing coronary angiography by BMI. (b) Unadjusted Kaplan Meier and 1-year cardiac-specific mortality in patients undergoing coronary angiography by BMI.

The unadjusted 1-year all-cause survival rates among the BMI categories revealed that the normal weight group had a higher mortality than the obese and overweight groups (*p* < 0.001) (Figure 2(a)). There were no significant differences among BMI categories for cardiac-specific mortality (*p* = 0.106) (Figure 2(b)).

Factors significantly associated with 1-year all-cause mortality during univariate analyses included age, HTN, diabetes, family history of premature CAD, CHF, PVD, CVD, COPD, malignancy, renal insufficiency, CRF, dialysis, DJS, and BMI as both categorical and continuous variable. The variables gender and hyperlipidemia were not significant. All statistically and clinically significant variables with *p* values < 0.20 were included in multivariate Cox proportional regression analysis. Significant correlates of 1-year all-cause mortality included age, diabetes, PVD, COPD, malignancy, renal insufficiency, DJSs 8, 10, and 12, and LV Grades III and IV. BMI was not a statistically significant correlate of all-cause mortality (Table 2). Cox regression analysis was also performed using BMI as a continuous variable; however, it was not a significant factor associated with 1-year all-cause mortality (data not shown).

Significant correlates of 1-year cardiac-specific mortality included age, CHF, DJSs 4 to 12, and LV Grades III and IV, but not BMI (Table 3).

## 4. Discussion

Our study examined the relationship between BMI and CAD and 1-year mortality in a large cohort of patients undergoing CA for suspected, but not yet confirmed, CAD. 84% of patients were overweight and obese. In the current study, obese patients were significantly younger (i.e., 3.7 years) than their nonobese counterparts and presented with less severe CAD based on DJSs despite having a higher prevalence of HTN, hyperlipidemia, and diabetes. Normal weight patients were older, had PVD, and had a history of renal insufficiency or were on dialysis.

Meta-analytic findings suggest a reverse J-shaped relationship between all-cause mortality and cardiovascular mortality and BMI in patients with established CAD [19, 20, 44]. However, very few studies have examined the association of BMI and CAD in patients undergoing CA for suspected, but unconfirmed, CAD. The current study findings support the findings of Rubinshtein et al. [29] and Niraj et al. [30]. Rubinshtein et al. [29] reported an inverse relationship between BMI and severity of CAD among 928 patients with CAD. Risk factors including diabetes, hyperlipidemia, and male gender were also correlated with severity of CAD. Niraj et al. [30] investigated the relationship between severity of CAD and BMI according to the DJS in a sample of 770 patients from the US. The authors also reported a paradoxical relationship. In both studies, obese patients were significantly younger than normal weight and overweight patients, leading to the conclusion that this association could be partly or completely explained by the increased likelihood of early physician referral of obese patients for cardiac catheterization and therefore at an earlier stage of CAD. The inverse relationship between BMI and severity of CAD was also reported most recently by Parsa and Jahanshahi [31] in a cross-sectional prospective study performed between September 2009 and March 2011 among 414 patients with suspected CAD undergoing CA.

After controlling for potential confounders such as other cardiovascular risk factors and comorbidities in our analyses, BMI did not emerge as an independent factor significantly associated with either all-cause or cardiac-specific mortality. Our study lacked the statistical power to support a potential doubling of cardiac-specific mortality or a 36% decrease among the obese group (HR 1.11, 95% CI .64–1.92).

It is important to note that, in the current study, significant proportions of overweight (39%) and obese (45.5%) patients who underwent CA did not have CAD based on angiographic generated DJSs. We were unable to examine the relationship between BMI and mortality in patients who had a CA but were not diagnosed with CAD due to low event rates (45 all-cause and 13 cardiac-specific deaths). An obesity paradox has been reported in patients who had CA with no CAD [19]. Two explanations were given for the unexpected finding: (1) other cardiac risk factors could classify these patients as having “preclinical” disease and that a higher BMI was protective and (2) referral and treatment bias in CAD since obesity is a “visible” risk factor that may predispose physicians to refer obese patients for CA earlier than those with a normal BMI. Niraj et al. [30] also suggested that the trend of normal or minimal change angiography in obese patients may have been due to a tendency of bias of physicians to refer obese patients for earlier angiography. Rubinshtein et al. [29] suggested that a younger age could be associated with a lower prevalence of high-risk coronary anatomy compared with nonobese older patients. This could partially explain the findings of the current study as well. Patients of normal weight were significantly older than their obese counterparts and had more angiographic severe CAD according to their DJSs.

Although the mechanism for the potential protective effect of obesity among patients with CAD remains unclear, a number of potential mechanisms have been proposed: greater metabolic reserves, less cachexia, younger presenting age, more aggressive medical therapy, more aggressive diagnostic and revascularization procedures, increased muscle mass and strength, possible improved cardiorespiratory fitness despite obesity, diminished hormonal response including the renin-angiotensin-aldosterone system, and unmeasured confounders, including selection bias [45]. It has been proposed that the apparent paradox that has been observed by other researchers may be the result of collider stratification, a source of selection bias that is common in epidemiology research [44]. According to Banack and Kaufman [46] the typical demonstration of this bias results from conditioning on a variable affected by exposure with the outcome (referred to as a collider). Distortion of the association between exposure and outcome as a result of this conditioning on a collider can therefore produce a spurious protective association between obesity and mortality in disease groups [46].

*Study Strengths and Limitations*. Our study has a number of strengths. We report on a large population-based cohort of consecutive patients undergoing CA at a single tertiary cardiac centre using APPROACH-NL prospectively collected data. Data quality assurance indicated that the amount of missing data was minimal (1.2%). Actual measures of weight and height were taken at the time of CA, unless the patients were unstable. In addition, although it is well documented that respondents have a tendency to underestimate their weight and/or overestimate their height, [47] self-reported height and weight are considered valid for identifying relationships in epidemiologic studies [48], with self-reported values being strongly correlated with measured values [49, 50]. We were able to assess the effect of BMI on 1-year all-cause and cardiac-specific mortality in patients with and without CAD using data linkage to up-to-date mortality data.

This study also has limitations. First, our study is an observational nonrandomized cohort study and therefore provides evidence of association not causation. Data from a clinical database was used and as such cannot account for potential residual or unmeasured confounders not captured in the database. Second, the study population was heterogeneous (i.e., included patients with variable levels of coronary artery disease severity ranging from acute coronary syndrome with cardiogenic shock to stable angina). Third, despite its widespread use, the use of BMI in terms of its accuracy to define obesity is controversial given its inability to differentiate lean mass and body fat [51–54]. BMI has been criticized as an inaccurate method to investigate body fatness because it is not as well correlated to CVD and death as other measures of obesity including waist circumference and waist-to-hip ratio [45], data that were unavailable in the APPROACH-NL clinical database. Fourth, BMI was collected at the time of the index CA only and potential changes in BMI were not accessed. Finally, this research examined BMI at an initial point in time and related it to mortality at 1 year. Comparisons were limited to three BMI groups: normal weight, overweight, and obese due to the relatively small sample size for patients and low event rates in the extreme ends of BMI classification.

## 5. Conclusions

This observational study failed to detect an association of BMI with 1-year all-cause or cardiac-specific mortality after adjustment for potential confounding variables.

## Figures and Tables

**Table 1:** Baseline characteristics of study subjects undergoing coronary angiography in relation to BMI category and Duke Jeopardy Score (DJS) based on coronary angiographic findings in relation to BMI category (*N* = 8079).

Variable	Normal *n* = 1297	Overweight *n* = 3072	Obese *n* = 3710	*p* value^*∗*^
Weight (kgs ± SD)	64.7 ± 8.8	78.8 ± 9.3	97.7 ± 16.0	0.000
BMI (mean ± SD)	22.9 ± 1.6	27.6 ± 1.4	35.0 ± 4.7	0.000
Age, years (mean ± SD)	63.4 ± 11.3	62.1 ± 10.6	59.7 ± 10.2	0.000
Male sex	744/1297 (57.4%)	2081/3072 (67.7%)	2249/3710 (60.6%)	0.000
HTN	735/1297 (56.7%)	1870/3069 (60.9%)	2629/3704 (71.0%)	0.000
Hyperlipidemia	991/1297 (76.4%)	2433/3068 (79.3%)	3022/3706 (81.5%)	0.000
Family history of premature CAD^*ǂ*^	730/1295 (56.4%)	1925/3063 (62.8%)	2421/3695 (65.5%)	0.000
Current/former smoker	889/1295 (68.6%)	2105/3060 (68.8%)	2557/3693 (69.2%)	0.890
Diabetes	203/1297 (15.7%)	638/3069 (20.8%)	1279/3708 (34.5%)	0.000
Renal insufficiency	67/1296 (5.2%)	124/3069 (4.0%)	130/3705 (3.5%)	0.030
Dialysis	16/1296 (1.2%)	15/3069 (0.5%)	19/3705 (0.5%)	0.009
CRF	38/1296 (2.9%)	69/3069 (2.2%)	75/3705 (2.0%)	0.166
Malignancy	67/1296 (5.2%)	132/3069 (4.3%)	153/3705 (4.1%)	0.282
COPD	210/1297 (16.2%)	398/3069 (13.0%)	697/3705 (18.8%)	0.000
PVD	91/1297 (7.0%)	131/3069 (4.3%)	139/3705 (3.8%)	0.000
CHF	25/1296 (1.9%)	54/3069 (1.8%)	81/3705 (2.2%)	0.450
CVD	90/1296 (6.9%)	163/3069 (5.3%)	204/3705 (5.5%)	0.088
ACS (*n* = 4359)	842 (19.3)	1709 (39.2)	1808 (41.5)	
STEMI	243 (28.9)	426 (39.2)	419 (38.5)	0.000
Non-STEMI	382 (45.4)	791 (46.3)	850 (47.0)	
Unstable angina	217 (25.8)	492 (28.8)	539 (29.8)	
LV grade				
I (>50%)	1067/1286 (83.0%)	2557/3033 (84.3%)	3154/3668 (86.0%)	0.003
II (35–50%)	136/1286 (10.6%)	334/3033 (11.0%)	353/3668 (9.6%)	
III (20–34%)	52/1286 (4.0%)	110/3033 (3.6%)	110/3668 (3.0%)	
IV (<20%)	31/1286 (2.4%)	32/3033 (1.1%)	51/3668 (1.4%)	
DJS				
≥2	771/1297 (59.4)	1875/3072 (61.0)	2023/3710 (54.5)	0.000
≥4	542/1297 (41.8)	1229/3072 (40.0)	1303/3710 (35.1)	0.000
≥6	424/1297 (32.7)	966/3072 (31.4)	992/3710 (26.7)	0.000
≥8	248/1297 (19.1)	56/3072 (18.5)	593/3710 (16.0)	0.006
≥10	162/1297 (12.5)	369/3072 (12.0)	395/3710 (10.6)	0.096
12	91/1297 (7.0)	198/3072 (6.4)	188/3710 (5.1)	0.010
Medications at time of referral				
Aspirin	1162/1297 (89.6)	2780/3071 (90.5)	3294/3709 (88.8)	0.071
Beta blockers	990/1295 (76.4)	2313/3071 (75.3)	2806/3709 (75.7)	0.729
ACE inhibitors	514/1295 (39.7)	1279/3071 (41.6)	1651/3708 (44.5)	0.004
ARB antagonists	116/1295 (9.0)	357/3071 (11.6)	663/3708 (17.9)	0.000
CCB	161/1295 (12.4)	446/3071 (14.5)	619/3708 (16.7)	0.000
Statin therapy	972/1296 (75.0)	2382/3071 (77.6)	2897/3709 (78.1)	0.068
LA nitrates	264/1295 (20.4)	629/3071 (20.5)	769/3709 (20.7)	0.951
Ticlopidine/clopidogrel	800/1297 (61.7)	1603/3071 (52.2)	1699/3708 (45.8)	0.000

Values are means ± SD or % (*n*/*N*).

ACE = angiotensin converting enzyme; ACS = acute coronary syndrome; ARB = angiotensin receptor blocker; CAD = coronary artery disease; CCB = calcium channel blockers; CHF = congestive heart failure; COPD = chronic obstructive pulmonary disease; CRF = chronic renal failure; CVD = cerebrovascular disease; DJS = Duke Jeopardy Score; HTN = hypertension; LA = long-acting; PVD = peripheral vascular disease.

^*ǂ*^Family history of CAD is positive if the patient has/had any direct blood relative (parent, siblings, and children) who have been diagnosed with angina, MI, or sudden cardiac death before age of 55 years.

*Note*. DJS, Duke Jeopardy Score, is a score from 0 to 12 which estimates the amount of myocardium at risk on the basis of particular location of stenosis. A score of 0 is indicative of a normal angiogram or noncritical (<70%) stenosis in any of the coronary arteries. A score of 0 was assigned to 526 (40.6%) normal weight patients, 1197 (39.0%) overweight patients, and 1687 (45.5%) obese patients.

^*∗*^*p* value for chi-square for categorical variables or ANOVA for continuous variables.

**Table 2:** Correlates of 1-year all-cause mortality calculated by Cox proportional hazards multiple regression analysis.

	Overall *n* = 8079	*β*	SE	Wald *χ*^2^	*p* value	HR	95% CI
Age	61.2 ± 10.6	.044	.008	26.802	0.000	1.04	1.03–1.06
DJS							
0 (referent category)	3410 (42.2%)			28.637	0.000		
2	1595 (19.7%)	−.021	.253	.007	0.932	.98	.60–1.61
4	692 (8.6%)	.296	.277	1.14	0.286	1.35	.78–2.31
6	973 (12.0%)	−.017	.286	.003	0.953	.98	.56–1.72
8	483 (6.0%)	.761	.268	8.088	0.004	2.14	1.27–3.62
10	449 (5.6%)	.662	.282	5.501	0.019	1.94	1.12–3.37
12	198 (6.4%)	.998	.239	17.415	0.000	2.71	1.70–4.33
BMI category							
Normal weight (referent category)	1297 (16.1)			4.213	0.122		
Overweight	3072 (38)	−.341	.189	3.255	0.071	.71	.49–1.03
Obese	3710 (45.9)	−.356	.194	3.37	0.066	.70	.48–1.02
LV Grade							
Grade I (referent category)	6778 (84.9)			50.146	0.000		
Grade II	823 (10.3)	.309	.216	2.038	0.153	1.36	.89–2.08
Grade III	272 (3.4)	1.226	.222	30.423	0.000	3.41	2.20–5.27
Grade IV	114 (1.4)	1.568	.280	31.337	0.000	4.80	2.77–8.31
Hypertension	5234 (64.9%)	−.040	.179	.050	0.823	.96	.68–1.37
Diabetes	342 (36.9%)	.175	.161	1.18	0.277	1.19	.87–1.63
Family history of premature CAD	5076 (63.0%)	−.170	.155	1.19	0.274	.84	.62–1.14
CHF	160 (2.0%)	.384	.259	2.193	0.139	1.47	.88–2.44
PVD	361 (4.5%)	.517	.223	5.361	0.021	1.68	1.08–2.60
CVD	457 (5.7%)	−.041	.232	.032	0.859	.96	.61–1.51
COPD	1305 (16.2%)	.585	.166	12.379	0.000	1.79	1.29–2.49
Malignancy	352 (4.4)	.559	.238	5.522	0.019	1.75	1.10–2.79
Renal insufficiency	321 (4.0%)	.666	.305	4.75	0.029	1.95	1.07–3.54
CRF	182 (2.3)	.355	.375	.898	0.343	1.43	0.89–4.28
Dialysis	50 (0.6)	.665	.402	2.738	0.098	1.95	0.68–2.97
Current/former smoker	5551 (69.0%)	.196	.174	1.26	0.262	1.22	.86–1.71

BMI = body mass index; CHF = congestive heart failure; CI = confidence interval; COPD = chronic obstructive pulmonary disease; CRF = chronic renal failure; CVD = cerebrovascular disease; DJS = Duke Jeopardy Score; HR = hazard ratio; LV = left ventricular; PVD = peripheral vascular disease; SE = standard error.

**Table 3:** Correlates of 1-year cardiac-specific mortality calculated by Cox proportional hazards multiple regression analysis.

	Overall	*β*	SE	Wald *χ*^2^	*p* value	HR	95% CI
	*n* = 8079						
Age	61.2 ± 10.6	.046	.012	14.646	0.000	1.05	1.02–1.07
DJS							
0 (referent category)	3410 (42.2%)			30.545	0.000		
2	1595 (19.7%)	.242	.436	.307	0.579	1.27	.54–3.00
4	692 (8.6%)	1.012	.421	5.763	0.016	2.75	1.20–6.28
6	973 (12.0%)	.941	.401	5.513	0.019	2.56	1.17–5.62
8	483 (6.0%)	1.46	.407	12.854	0.000	4.31	1.94–9.57
10	449 (5.6%)	1.245	.432	8.317	0.004	3.47	1.49–8.09
12	198 (6.4%)	1.762	.367	23.023	0.000	5.83	2.84–11.99
BMI category							
Normal weight (referent category)	1297 (16.1)			2.72	0.257		
Overweight	3072 (38)	−.305	.293	1.083	0.298	.74	.42–1.31
Obese	3710 (45.9)	.100	.284	.126	0.722	1.11	.64–1.92
LV grade							
Grade I (referent category)	6778 (84.9)			37.607	0.000		
Grade II	823 (10.3)	.239	.320	.559	0.455	1.27	.68–2.38
Grade III	272 (3.4)	1.154	.320	12.987	0.000	3.17	1.69–5.94
Grade IV	114 (1.4)	1.98	.348	32.352	0.000	7.24	3.66–14.33
Hypertension	5234 (64.9%)	.112	.274	.167	0.682	1.12	.65–1.91
Diabetes	342 (36.9%)	.312	.225	1.93	0.165	1.37	.88–2.12
Family history of premature CAD	5076 (63.0%)	−.098	.222	.196	0.658	.91	.59–1.40
CHF	160 (2.0%)	.725	.340	4.56	0.033	2.07	1.06–4.02
PVD	361 (4.5%)	.510	.322	2.512	0.113	1.67	.89–3.13
CVD	457 (5.7%)	−.027	.329	.007	0.935	.97	.51–1.89
COPD	1305 (16.2%)	.275	.247	1.238	0.266	1.32	.81–2.14
Malignancy	352 (4.4)	−1.88	1.01	3.491	0.062	.150	.02–1.10
Renal insufficiency	321 (4.0%)	.466	.448	1.085	0.298	1.59	.66–3.83
CRF	182 (2.3)	.2925	.544	.288	0.591	1.34	.46–3.89
Dialysis	50 (0.6)	.422	.636	.440	0.507	1.53	.44–5.31
Current/former smoker	5551 (69.0%)	.272	.255	1.14	0.286	1.31	.86–1.71

BMI = body mass index; CHF = congestive heart failure; CI = confidence interval; COPD = chronic obstructive pulmonary disease; CRF = chronic renal failure; CVD = cerebrovascular disease; DJS = Duke Jeopardy Score; HR = hazard ratio; LV = left ventricular; PVD = peripheral vascular disease; SE = standard error.
